# Implications of T cell-mediated tumor killing genes for molecular heterogeneity and clinical stratification in lung adenocarcinoma

**DOI:** 10.1016/j.gendis.2023.101162

**Published:** 2023-11-08

**Authors:** Tong Qiao, Beining Yin, Jun Liang, Li Wei

**Affiliations:** a Department of Thoracic Surgery, Henan Provincial People's Hospital, Zhengzhou, Henan 450000, China; b Reproductive Medicine Center, The First Affiliated Hospital of Zhengzhou University, Zhengzhou, Henan 450052, China; c Department of Breast Surgery, Henan Provincial People's Hospital, Zhengzhou, Henan 450000, China

Due to the profound heterogeneity exhibited amongst patients with lung adenocarcinoma (LUAD), considerable variances in clinical efficacy emerge.^1^ For the aims of precision medicine, a clinical instrument to delineate distinct disease phenotypes and anticipate susceptibility to intervention is imperative. Cellular cytotoxicity mediated through T lymphocytes constitutes a pivotal mechanism of anti-tumoral immunity and the foundation for cancer immunotherapy.^2^ Numerous genes regulating the sensitivity of tumor cells to killing mediated by T lymphocytes (GSTKKs) are conducive to satisfying the aforesaid medical necessity.

A total of 1735 samples across four independent cohorts were enrolled, including the TCGA-LUAD cohort with complete clinical information and three GEO cohorts for external validation. Premised upon significant differential expression between tumor and normal tissues (Fig. S1A) and correlation with patient overall survival in univariate Cox regression analysis (Fig. S1B), 32 GSTKKs appeared notably correlated with prognosis (*P* < 0.01; Fig. S1C). Consensus cluster analysis founded on expression levels of the 32 GSTKKs signature delineated three stable sub-phenotypes termed GSTKK1, GSTKK2, and GSTKK3 (Fig. S2, S3A–C). Moreover, UMAP analysis also indicated clustering into the three sub-phenotypes could effectively individualize the examples (Fig. S3D). To achieve more precise stratification of patients, samples with silhouette coefficients exceeding 0 were screened in further analyses (Fig. S3E).

Molecular subtypes lacking clinical implications have restricted applicability. Hence, we evaluated associations between the GSTKKs-based subtypes and patient outcomes. Kaplan-Meier analysis uncovered significant survival disparities among the three subtypes in TCGA-LUAD (*n* = 458, *P* < 0.001; Fig. S3F). Patients classified as GSTKK2 exhibited the most dismal prognosis with a median overall survival of 25 months, contrasted with 45 months for GSTKK1 and 35 months for GSTKK3. Analogous conclusions were obtained in three validation cohorts from discrete gene expression platforms, including GSE68465 (*n* = 420, *P* = 0.0005), GSE72094 (*n* = 385, *P* < 0.0001), and GSE31210 (*n* = 216, *P* = 0.0035) (Fig. S4A–F), underscoring reproducibility. These revelations intimated that the classifier founded on prognostic GSTKKs not only encompassed molecular heterogeneity but also delineated patient subgroups with markedly variable clinical trajectories.

To probe the biological heterogeneity fundamental to the subtypes, we executed gene set enrichment analysis (GSEA) to pinpoint pathways disparately activated across subtypes. The GSTKK2 subtype was enriched for cell cycle progression pathways like reactive oxygen species and mitochondrial metabolism, harmonious with a highly proliferative phenotype. Targeting these conduits could furnish potential therapeutic strategies for this disorder. GSTKK3 was typified by robust enrichment of pathways associated with immune activation like IL6-JAK-STAT signaling, TNF-α signaling, and IFN-γ response (Fig. S5A). GO enrichment analysis emphasized biological processes allied to cell proliferation also tallied higher in the GSTKK2 subtype (Fig. S5B, 6A). Multifarious studies have evinced that the specific marker *MKI67* exerted a substantial role in LUAD evolution, particularly in bolstering tumor growth and metastasis.^3^ The maximal *MKI67* expression in the GSTKK2 subtype could also exemplify the most ominous prognosis (Fig. S5C). These discoveries provided initial evidence that the LUAD subtypes delineated by prognostic GSTKKs encompass heterogeneity in tumor-immune interplays and intrinsic disease biology.

The mutational heterogeneity of the three subtypes was probed by analyzing frequencies of somatic mutations, copy number variations, and metrics of genomic instability (Fig. S6B, 7A). Most frequent driver mutations were enriched in GSTKK2 compared with other subtypes (Fig. S7B). Mutations in genes like *TP53* and *TTN* have been pinpointed as cardinal drivers of LUAD, and the accumulation of additional genetic mutations could further contribute to tumor growth and progression.^4^ An in-depth examination of the roles of these mutated genes in LUAD could be highly consequential for accurate classification and targeted therapy of patients. Measures of genomic instability, including the fraction of genome altered, fraction of genome gained, and fraction of genome lost, were elevated in GSTKK2 versus other subtypes. Notably, for both amplifications and deletions, the GSTKK2 subtype exhibited the highest burden at the chromosomal arm and segment levels, harmonious with the mutation landscape (Fig. S7C). Further appraisal of the mutation landscape revealed GSTKK2 had higher tumor mutational burden, aneuploidy score, and homologous recombination deficiency (Fig. S7D–F). Collectively, these conclusions underscore that an unstable, mutation-rich phenotype typified GSTKK2. The high mutational and copy number variation burden of GSTKK2 implied opportunities for targeted therapy against frequent driver events and a greater likelihood of treatment resistance.

To ascertain optimal clinical treatment regimens, it was indispensable to probe the heterogeneity of each subtype regarding the immune landscape. GSTKK3 displayed the most extensive immune infiltrates, including elevated CD8^+^ T cells, natural killer cells, and dendritic cells, intimating it could recruit ample immune cells (Fig. S8A). Research has established that elevated levels of tumor-infiltrating lymphocytes are associated with enhanced survival in non-small cell lung cancer patients.^5^ Additionally, the maximal leukocyte fraction denoted GSTKK3 exhibited a more forceful response to immunotherapy, harmonious with an inflamed phenotype (Fig. S8B). In contrast, only activated CD4 T cells were significantly enriched in the GSTKK2 subtype. Thus, GSTKK2 was regarded as an immune-cold tumor (Fig. S8C). Similarly, most immune checkpoints were highly expressed in GSTKK3, indicating immunotherapy could have favorable efficacy for GSTKK3 patients (Fig. S6C, 6D, 8D). Due to the lowest expression of antigen-presenting molecules and antigen presentation machinery score in GSTKK2, it was speculated these patients could lack effective antigen presentation, resulting in failed recruitment of immune cells to destroy tumor cells, resulting in poor prognosis (Fig. S8E, F).

Further cancer immune cycle analysis (Fig. 1A) also demonstrated immunotherapy could be more apposite for GSTKK3 patients, concordant with its traits (Fig. 1B). Notably, tumor characteristics of the GSTKK1 subtype were nearly absent, and the tumor inflammation score in GSTKK1 was the lowest, which intimated GSTKK1 had less tumor cell invasion and aligned with the most favorable prognosis (Fig. 1C). To further gauge the predictive value of the subtypes for immunotherapy response, we utilized *Submap* to deduce sensitivity to immune checkpoint inhibitors anti-PD1 and anti-CTLA4 founded on tumor immune microenvironment features of each subtype. Subsequent discoveries revealed individuals with the GSTKK3 subtype exhibited an enhanced response to immunotherapy and could potentially experience improved clinical outcomes (Fig. 1D).

**Figure 1:**
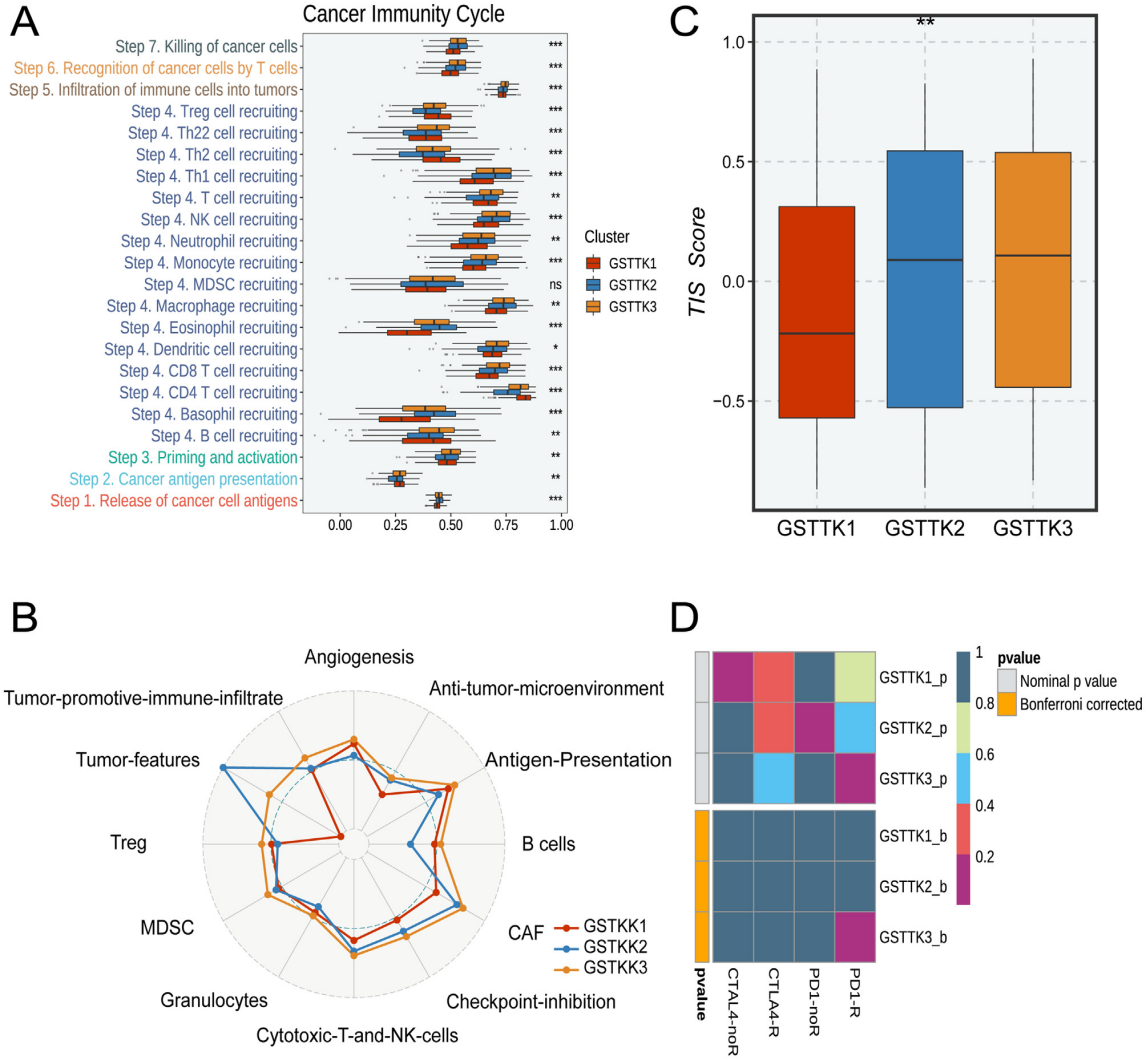
Characteristics of immune circulation GSTKKs-based subtypes. **(A)** Enrichment scores for the seven anti-tumor immune cycle steps were calculated with the ssGSEA algorithm. *P* values are shown as ^∗^*P* < 0.05, ^∗∗^*P* < 0.01, ^∗∗∗^*P* < 0.001, and ^∗∗∗∗^*P* < 0.0001. **(B)** The comparison of T-cell inflammatory signature (TIS) prediction scores among GSTKKs-based subtypes. **(C)** The radar map displayed the proportion of the immune-related characteristics and immune molecules in the three subtypes. **(D)** Submap analysis of the three subtypes with detailed anti-PD1 and anti-CTLA4 therapy information.

In summary, this study exploited prognostic GSTKKs to delineate LUAD subtypes with distinct clinical outcomes, biology characteristics, mutation landscapes, immune traits and predicted therapeutic responses. It furnished a framework for tailored therapy contingent on subtype-specific targets and immunotherapy sensitivity. These discoveries demonstrated the capability of predictive biomarkers to guide precision oncology through molecular subtyping.

## Author contributions

Tong Qiao and Li Wei: conceptualization, data curation, formal analysis, investigation, methodology, resources, software, validation, visualization, and writing-original draft. Tong Qiao and Beining Yin: conceptualization, project administration, supervision, and writing - review & editing. Tong Qiao, Jun Liang, and Beining Yin: writing review & editing. Tong Qiao, Beining Yin, Jun Liang, and Li Wei: verification of the underlying data. All authors read and approved the final version of the manuscript.

## Conflict of interests

The authors declare that they have no competing interests.

## Figures and Tables

**Figure:**
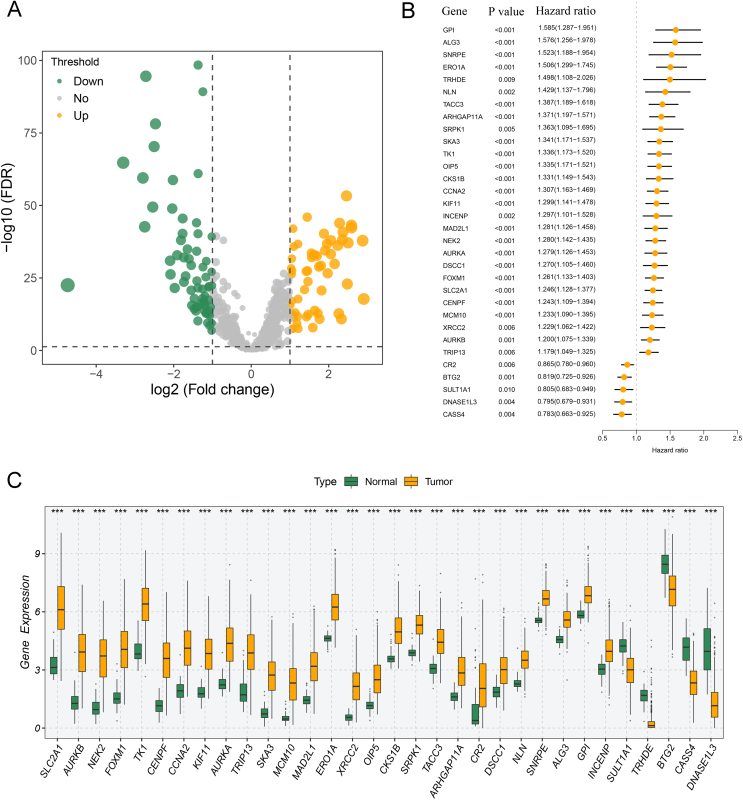
Figure S1 Identification of the prognostic-associated GSTKKs.

**Figure:**
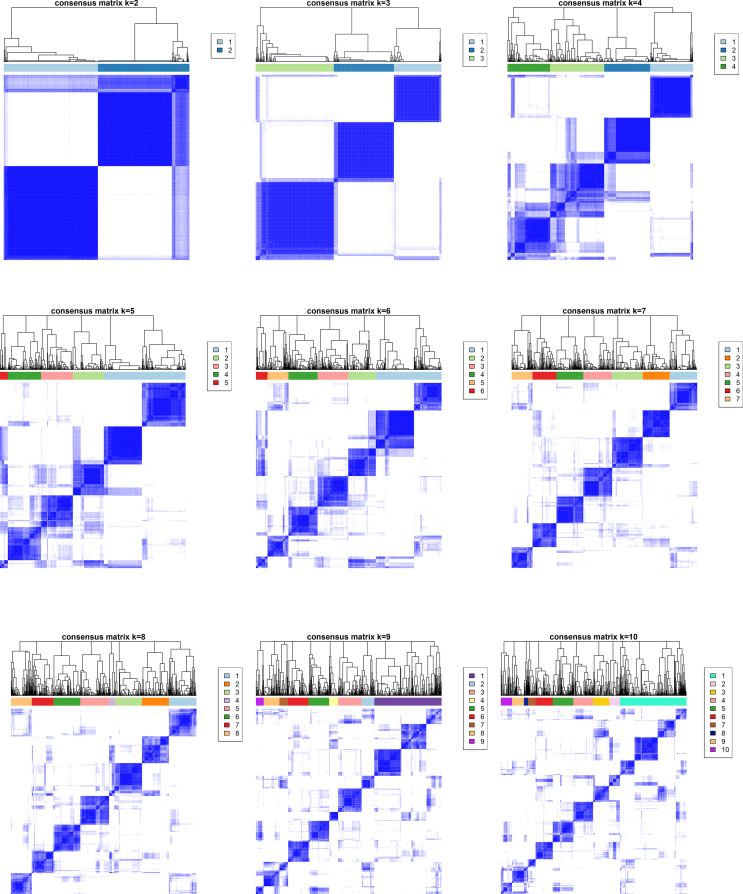
Figure S2 The consensus score matrix of GSTKKs-based subtypes using consensus unsupervised clustering with different *K* values.

**Figure:**
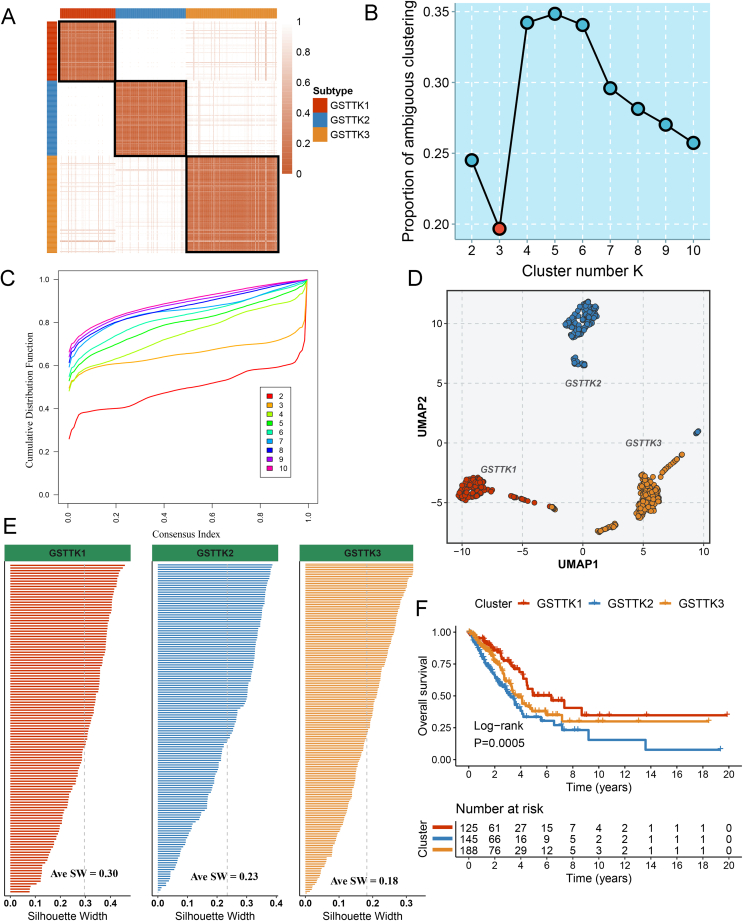
Figure S3 Identification of GSTKKs-based subtypes.

**Figure:**
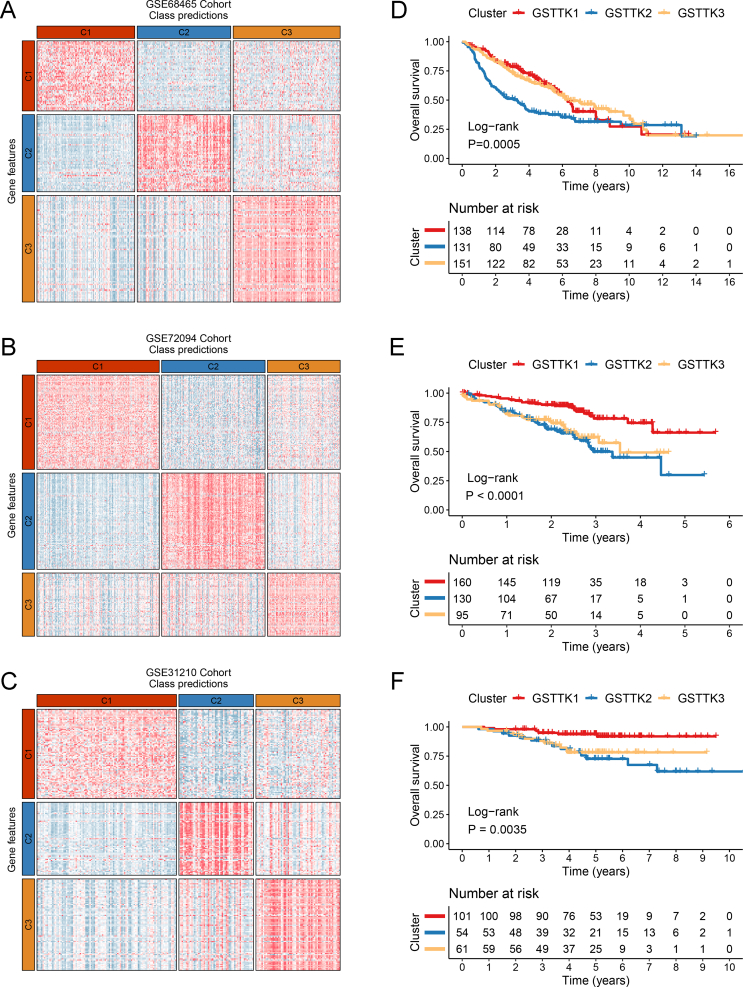
Figure S4 Validation of GSTKKs-based subtypes in three independent cohorts by the nearest template prediction (NTP) approach.

**Figure:**
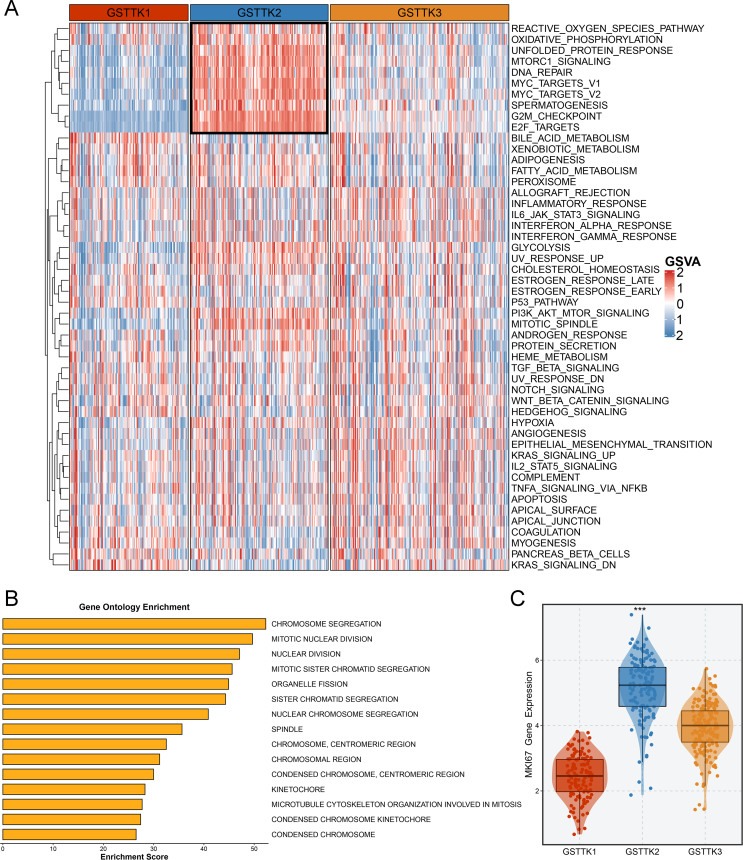
Figure S5 Biological characteristics of the three subtypes.

**Figure:**
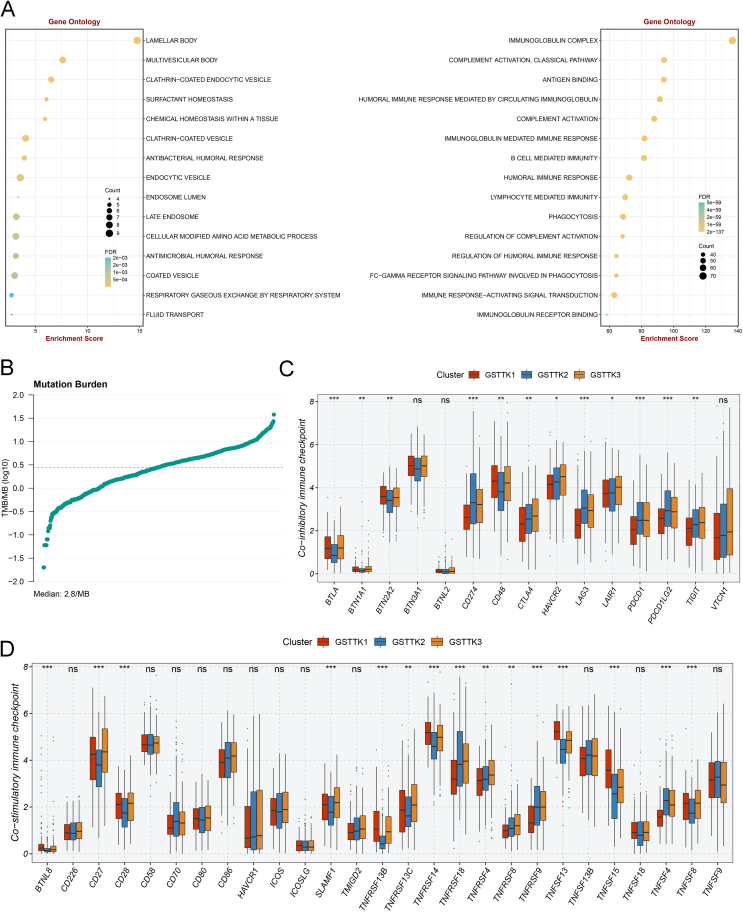
Figure S6 Gene ontology of GSTKK2 and the proportion of immune cells in the three subtypes.

**Figure:**
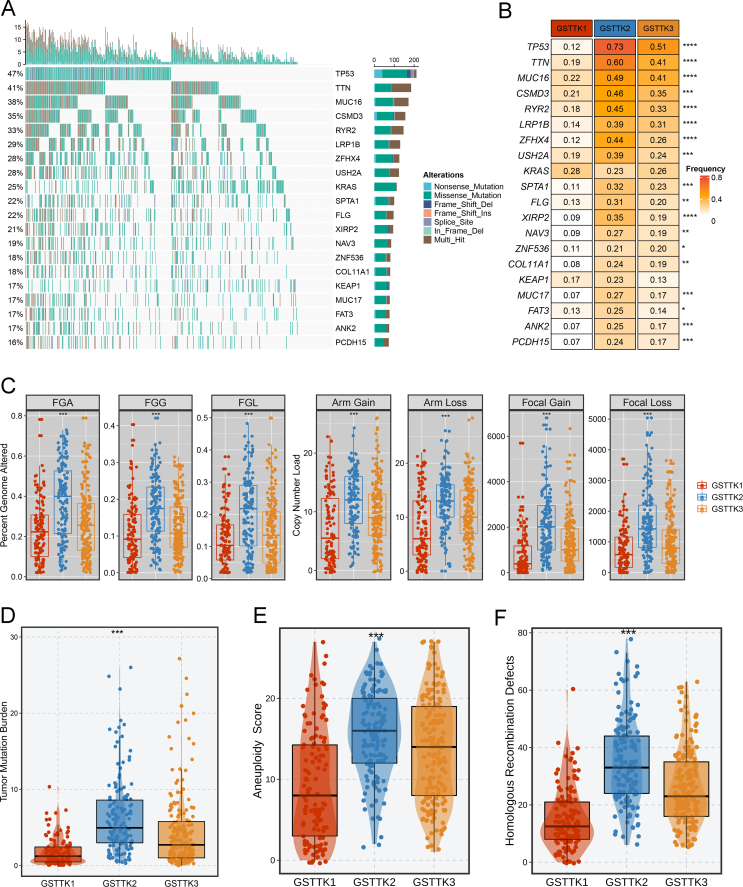
Figure S7 The mutational landscape of the three subtypes.

**Figure:**
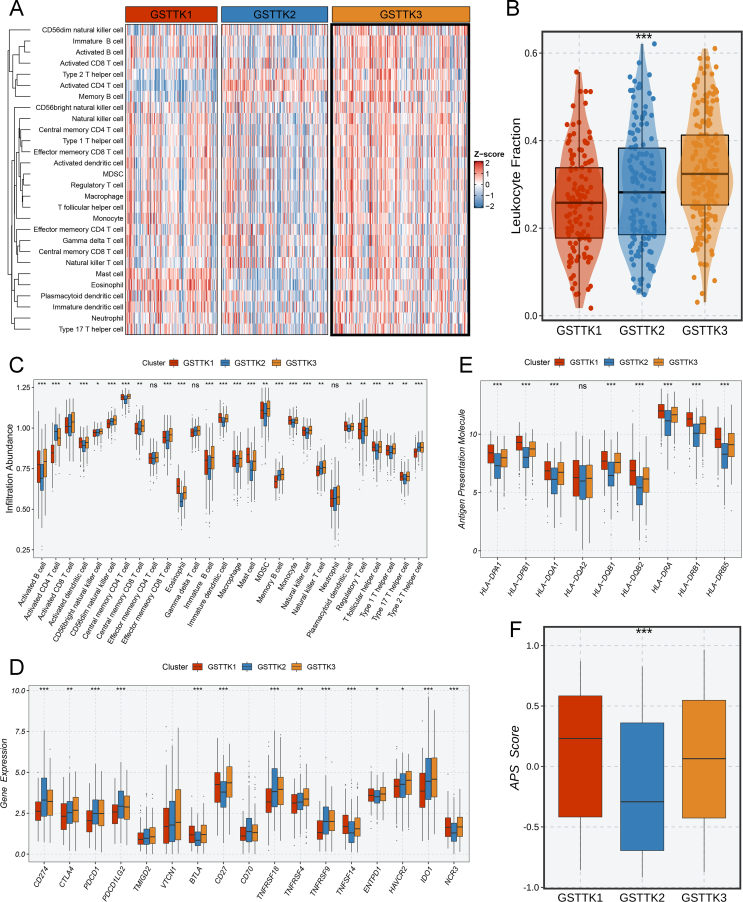
Figure S8 Analysis of the proportion of immune cells in the three subtypes.
