# Supplementary material for: Implications of T cell-mediated tumor killing genes for molecular heterogeneity and clinical stratification in lung adenocarcinoma
Source: Genes Dis. 2023 Nov 8;11(4):101162. doi: 10.1016/j.gendis.2023.101162 (PMC10914415; doi:10.1016/j.gendis.2023.101162)
Supplement: Multimedia component 1 [file mmc1.docx]

**Supplemental Figures Legends:**

**Figure S1. Identification of the prognostic-associated GSTKKs. (A).**The distribution of DEGs was shown in the volcano plot. **(B).**Univariate Cox regression analysis of the prognostic-associated GSTKKs. **(C).**The prognostic-related GSTKKs expression levels in tumor and normal tissues.

**Figure S2. The consensus score matrix of GSTKKs-based subtypes using consensus unsupervised clustering with different *K* value.**

**Figure S3. Identification of GSTKKs-based subtypes. (A).**The consensus score matrix of GSTKKs-based subtypes using consensus unsupervised clustering. **(B).**The proportion of ambiguous clustering (PAC) score, the lowest PAC value often means the optimal *K* value (*k=3*). **(C).**The cumulative distribution functions of consensus matrix for each *k* (indicated by colors). **(D).**The UMAP algorithm displayed the two-dimensional principal component diagram of three subtypes, with each point representing a single sample. **(E).**The silhouette statistic of three GSTKKs-based subtypes. **(F).**Kaplan-Meier curve of OS according to GSTKKs-based subtypes in TCGA-LUAD cohort.

**Figure S4. validation of GSTKKs-based subtypes in three independent cohorts by the nearest template prediction (NTP) approach. (A-C).** Verification of the three GSTKKs-based subtypes in GSE68465, GSE72094, and GSE31210 cohorts with NTP analysis. **(D-F).** Kaplan-Meier curves of OS according to the GSTKKs-based subtypes in GSE68465, GSE72094, and GSE31210 datasets, and proportion of patients in the subtypes.

**Figure S5. Biological characteristics of the three subtypes. (A).** Heatmap based on the score of each subtype in 50 Hallmark gene sets. The higher the score, the higher the pathway activity. **(B).** The Gene Ontology (GO) enrichment score of GSTKK2 subtype. **(C).** Expression levels of the LUAD marker *MKI67* gene in the three subtypes.

**Figure S6. Gene ontology of GSTKK2 and the proportion of immune cells in three subtypes. (A).** The gene ontology (GO) enrichment score in GSTKK2 subtype. **(B).** The plot of the Mutation burden, Median: 2.8/MB. **(C-D).** Expressions of Co-inhibitory immune checkpoints **(C)** and Co-stimulatory immune checkpoints **(D)** among GSTKKs-based subtypes.

**Figure S7. The mutational landscape of three subtypes. (A).** Mutation landscape of top 20 frequently mutated genes (FMGs) in the three clusters. **(B).** The mutation frequency of top 20 FMGs among three subtypes. **(C).** The differences of fraction of genome altered (FGA), fraction of genome gained (FGG), fraction of genome lost (FGL) in clinical characteristics ,and the burden of copy number gain or loss in arm and focal levels. *P* values are shown as **P < 0.05*; ***P < 0.01*; ****P < 0.001*. **(D-F).** Comparison of tumor mutation burden (TMB), aneulpoidy score, and homologous recombination defects (HRD) levels of three subtypes.

**Figure S8. Analysis of the proportion of immune cells in three subtypes. (A).** The heatmap illustrated the correlation between three subtypes and clinical characteristics, and the infiltration abundance of 28 immune cell subsets evaluated by ssGSEA algorithm. **(B).** The comparison of leukocyte fraction among GSTKKs-based subtypes. **(C).** Infiltration abundance of immune cells among the three subtypes. *P* values are shown as **P < 0.05*, ***P < 0.01*, ****P < 0.001*, *****P < 0.0001*, and ns was the abbreviation of no significance. **(D-E).** Expressions of checkpoint genes and HLA moleculars among GSTKKs-based subtypes. **(F).** The comparison of antigen processing and presenting machinery score (APS) in three subtypes.
